# Improving the Stability of High-Voltage Lithium Cobalt Oxide with a Multifunctional Electrolyte Additive: Interfacial Analyses

**DOI:** 10.3390/nano11030609

**Published:** 2021-02-28

**Authors:** Xing-Qun Liao, Feng Li, Chang-Ming Zhang, Zhou-Lan Yin, Guo-Cong Liu, Jin-Gang Yu

**Affiliations:** 1 College of Chemistry and Chemical Engineering, Central South University, Changsha 410083, China; xqliao@highpowertech.com; 2 Research Institute of Highpower International, Huizhou 516057, China; fli@highpowertech.com (F.L.); maxwell.zhang@highpowertech.com (C.-M.Z.); 3 School of Chemistry and Materials Engineering, Huizhou University, Huizhou 516007, China

**Keywords:** high-voltage, lithium cobalt oxide, multifunctional electrolyte additives, interfacial stability

## Abstract

In recent years, various attempts have been made to meet the increasing demand for high energy density of lithium-ion batteries (LIBs). The increase in voltage can improve the capacity and the voltage platform performance of the electrode materials. However, as the charging voltage increases, the stabilization of the interface between the cathode material and the electrolyte will decrease, causing side reactions on both sides during the charge–discharge cycling, which seriously affects the high-temperature storage and the cycle performance of LIBs. In this study, a sulfate additive, dihydro-1,3,2-dioxathiolo[1,3,2]dioxathiole 2,2,5,5-tetraoxide (DDDT), was used as an efficient multifunctional electrolyte additive for high-voltage lithium cobalt oxide (LiCoO_2_). Nanoscale protective layers were formed on the surfaces of both the cathode and the anode electrodes by the electrochemical redox reactions, which greatly decreased the side reactions and improved the voltage stability of the electrodes. By adding 2% (wt.%) DDDT into the electrolyte, LiCoO_2_ exhibited improved Li-storage performance at the relatively high temperature of 60 °C, controlled swelling behavior (less than 10% for 7 days), and excellent cycling performance (capacity retention rate of 76.4% at elevated temperature even after 150 cycles).

## 1. Introduction

In the past decades, remarkable progress has been made in lithium-ion batteries (LIBs) in the field of portable devices [1,2]. In addition, LIBs can be used as efficient aerospace batteries for small satellites, and LIBs are becoming one of the most widely used energy storage devices due to their relatively high working potential and high energy density [3,4,5,6]. However, it is well known that the energy density of cathode materials is the main factor affecting the performance of LIBs. To meet the requirements of higher capacity and longer cycle life for LIBs, there has been an increase in studies focusing on the development of novel cathodes with higher working potentials [7,8,9]. However, the overcharge of LiCoO_2_ can provoke the serious oxidation of the electrolyte at higher potentials and cause a high-resistance cathodic film to form, thus causing the capacity fade of the LIBs in the following cycles [10,11,12]. Some side effects have been observed, including the dissolution of transition-metal ions in the electrolyte and the reduced cycling stability of the cells [13]. It is well known that the conventional organic carbonate solvents have oxidization potentials of 5 V. In addition, the oxidation reaction is catalyzed in the presence of transition-metal ions, and the decomposition of electrolytes is accelerated at lower potentials, leading to unexpected rapid capacity fading [14]. Ethers such as 1,3-dioxolane (DOL) and 1,2-dimethoxyethane (DME) also have high ionic conductivities and coulombic efficiencies [15,16]. However, ethers at typical salt concentrations of 0.1 or 1 M cannot be practically utilized because of their low oxidative stability (less than 4 V vs. Li/Li^+^) [16,17,18]. Sulfone-based high-voltage electrolytes with good oxidation resistance have low lattice energy, but their relatively greater wettability and higher viscosity can greatly affect the performances of LIBs [19]. For cell systems containing graphite negative electrodes, sulfone-based electrolytes are also restricted since the stable solid–electrolyte interface (SEI) at the graphite surface cannot be formed [20]. Compared with organic carbonate solvents, room-temperature ionic liquids (RTILs) have exhibited higher thermal stability, lower flammability and volatility, and wider electrochemical windows [21]. However, the compatibilities of these electrolytes are also unsatisfactory due to the low wettability. In addition, the relatively higher melting points have also been found to degrade their low-temperature performances [22].

Electrolytes with excellent electrochemical properties always play an important role in improving the stability of LIBs. However, researchers have had great difficulty in developing novel electrolytes for high-performance LIBs. It is worth noting that the addition of a small amount of additive into the electrolyte is beneficial in forming a protective layer and preventing the solvent penetration; in this way, the possible damages to the electrode structure could be efficiently avoided. In addition, a film-forming additive for high-voltage cathode material in LIBs undergoes oxidation and decomposition reactions on the surface of the positive electrode, and a stable interface film is formed favoring the solvent system, thus reducing or preventing the further oxidation of the solvent system. Lithium bis(oxalato)borate (LiBOB) is a typical inorganic additive for high-voltage LIBs. Phosphides (such as tris(pentafluorophenyl)phosphine (TPFPP), tris(hexafluoro-iso-propyl)phosphate (HFiP) and N-(triphenylphosphoranylidene)aniline (TPPA)), sulfonate esters (such as methylene methanedisulfonate (MMDS)), carboxyl anhydrides (such as glutaric anhydride and succinic anhydride), and fluorides (such as 1,1-difluoro-4-phenylbut-1-ene (DF)) exhibited similar surface-film-forming characters and could enhance the performance of high-voltage cathodes [23,24,25,26,27,28,29]. The oxidation of LiBOB on the cathode surface was found to generate a cathode passivation layer that inhibited the further oxidation of the electrolyte [30,31].

The complexing additives can form complexes with free transition-metal elements, purify the electrolyte system, suppress the electrolyte decomposition, and improve the high-voltage performance. For example, adiponitrile can inhibit the side reaction between the electrolyte and the surface of the high-nickel positive electrode, and the strong coordination between the nitrile group and Ni^4+^ can effectively reduce the formation of electrochemically inert NiO-type rock-salt structure [32].

Recently, dihydro-1,3,2-dioxathiolo[1,3,2]dioxathiole 2,2,5,5-tetraoxide (DDDT) has emerged as an efficient electrolyte additive for LIBs. DDDT has been utilized as an overall-functional electrolyte additive for high-voltage NCM523/graphite batteries, and enhanced electrochemical performance could be obtained [33]. In this study, to improve the interface stability of high-voltage lithium cobalt oxide (LiCoO_2_), DDDT-containing electrolyte was used as a multifunctional electrolyte additive. The physicochemical properties of the cells were analyzed, and the underlying mechanisms were investigated. In particular, the protective layers formed electrodes by the electrochemical redox reactions on the surfaces of both the cathode and the anode could greatly decrease the side reactions and improve the voltage stability of the electrodes. The research indicated that the DDDT-containing electrolyte was beneficial for the high-voltage LiCoO_2_ batteries, besides the previous breakthroughs towards LiNi_0.5_Co_0.2_Mn_0.3_O_2_/graphite batteries, which may provide a useful reference for the preparation of more stable LIBs by the formation of high-quality interfacial films in the cells.

## 2. Experimental Details

### 2.1. Materials, Electrolyte Configuration, and Cell Production

**Figure 1:**
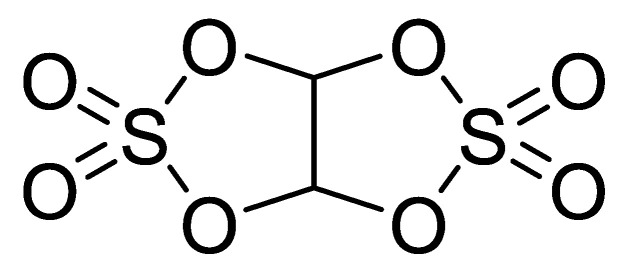
The chemical structure of dihydro-1,3,2-dioxathiolo[1,3,2]dioxathiole 2,2,5,5-tetraoxide (DDDT).

Battery-level component LiPF_6_, ethylene carbonate (EC), propylene carbonate (PC), diethyl carbonate (DEC), ethyl methyl carbonate (EMC), n-propyl propionate (PP), and dihydro-1,3,2-dioxathiolo[1,3,2]dioxathiole 2,2,5,5-tetraoxide (DDDT, Figure 1) were provided by Shanshan New Materials (Quzhou) Co., Ltd. The solvent was fully dried by 4A molecular sieve, activated, and configured in an argon (Ar)-filled glove box with water and oxygen below 1 ppm. The electrolyte model is shown in Table 1 and Table 2. The moisture and free acid contents of the electrolyte were tested by Metrohm Coulomb Karl Moisture Meter (below 20 ppm) and confirmed by triethylamine titration (below 50 ppm).

The cathode formulation consisted of 95 wt.% LiCoO_2_ (provided by Tianjin Bamo Tech Co. Ltd.; Tianjin, China), 2.5 wt.% carbon black, and 2.5 wt.% PVDF. A slurry with a viscosity of about 6000 mPa∙s was prepared by dispersing and mixing LiCoO_2_ and the conductive agent with NMP. By an aluminum foil current collector with the slurry, a positive electrode was obtained after drying. A conductive agent consisting of 95 wt.% graphite (Jiangxi Zichen Tech Co., Ltd.; Yichun, China), 2.5 wt.% carbon black, 1.5 wt.% SBR, and 1 wt.% CMC was used. By dispersing and mixing active materials and the conductive agent with deionized water to form a slurry with a viscosity of about 3000 mPa∙s and then coating the copper foil current collector with the slurry, a negative electrode was obtained after drying. The polypropylene separator was obtained from Shenzhen Senior Technology Material Co., Ltd. (Shenzhen, China). The negative electrode had an active mass load of approximately 1.07 mg cm^−2^, and the positive electrode had an active mass load of approximately 1.86 mg cm^−2^. A three-electrode system with Pt metal as the working electrode, lithium metal as the counter electrode, and a reference electrode was used for linear scan voltammetry (LSV). The potential of the electrode was scanned from open-circuit voltage (OCV) to 7.0 V, and the scanning speed was 0.1 mV/s. Cyclic voltammetry (CV) tests of the Li/graphite half cells were performed, and a scanning voltage range of 3–0.01 V was investigated with a sweep rate of 0.01 mV/s. The cells were measured by an eight-channel Solartron potentiostat (model 1470E; Advanced Measurement Technology Inc.; Oak Ridge, TN, USA).

To evaluate the reaction window of the fabricated electrolyte, we assembled the positive and negative electrodes and a separator in a CR2032 coin cell battery. Lithium metal was used as the reference electrode, and model #1–#4 electrolytes were utilized (Table 1). In order to evaluate the electrical performance of the additive on the full battery, we assembled the positive and negative pole pieces and the separator into a wound type 404,798 battery by using model #5–#8 electrolytes (Table 2).

The surface morphologies of the electrodes before and after cycle tests were investigated by a scanning electron microscope (SEM; Nova Nano SEM450). The functional groups of the formed cathode–electrolyte interface (CEI) layers before and after cycles, as well as their chemical compositions, were confirmed by an X-ray photoelectron spectroscope (XPS; R3000, VG SCIENTA). The crystal forms of the electrode materials before and after cycle tests were investigated by X-ray powder diffraction (XRD; D8 ADVANCE).

### 2.2. Electrochemical Properties

Firstly, the analyses of dQ/dV curves were conducted to quantitatively evaluate the performance of the full battery, and the high-temperature storage and the cycle characteristics were also evaluated. The voltages ranged from 3.0 to 4.5 V. Secondly, the AC impedance of the coin cells, i.e., the LiCoO_2_/Li and graphite/Li systems, which charge and discharge were tested after the assembled cells were charged and discharged at 0.2 C for one cycle, respectively. Impedance data were collected in the frequency range 0.01–100,000 Hz with the amplitude of 5 mV. High-temperature storage tests were carried out at 60 °C for three cells per group, and the hot thickness was tested regularly. The recharging currents of 0.7 C (overall cycle 250 and 150 times) were tested at 25 and 45 °C, and the internal resistance of the battery, or the discharge capacity retention (DCR), was tested at 25 °C.

## 3. Results and Discussion

### 3.1. Influence of DDDT on the Electrochemical Window

**Figure 2:**
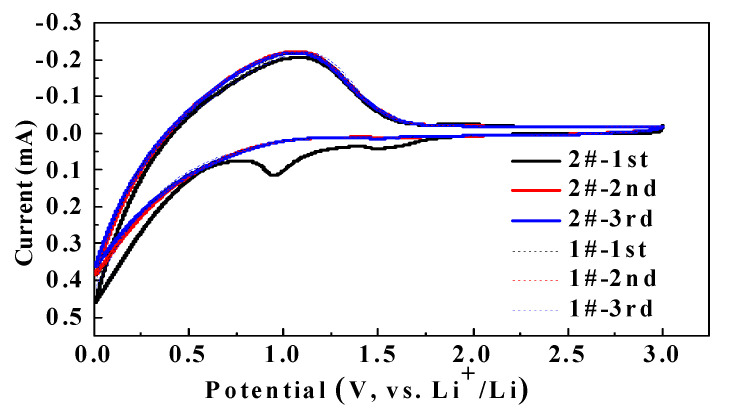
The CV plots for the first three cycles of the Li/graphite half cells without additive and with 0.5% DDDT.

Figure 2 shows the CV results for the Li/graphite button-type half cells. Comparing the cells in #1 and #2 electrolytes, it can be found that the redox peak potential of the cell in #2 electrolyte containing 0.5% DDDT appeared at 1.0 V during the first cycle, while it disappeared during the second and the third cycles. We speculated that the reduction reaction occurred during the first cycle due to the addition of DDDT into the electrolyte, which prevented further reaction due to the formation of SEI on the graphite electrode.

**Figure 3:**
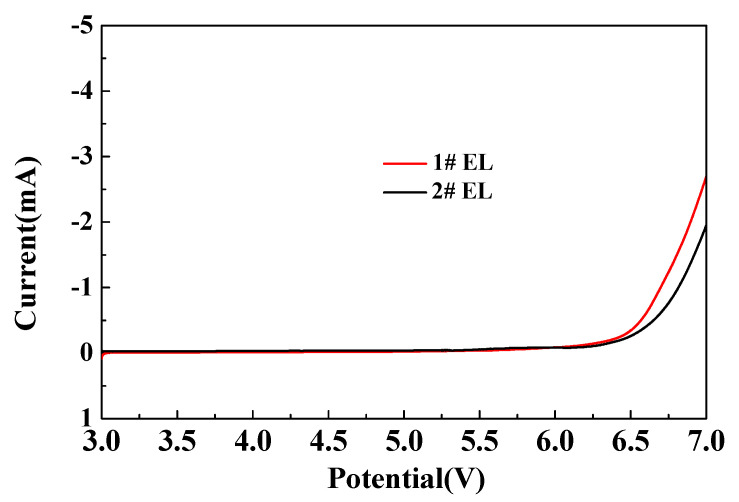
The LSV plots of the different electrolytes without additive and with 0.5% DDDT over a voltage range from the open-circuit voltage (OCV) to 7.0 V.

Figure 3 shows the results of the linear sweep voltammetry (LSV) of the Li/graphite button-type half cells. Comparing the cell in #1 electrolyte with that in #2 electrolyte, it is obvious that the former showed a higher current than the latter at above 6.5 V. We speculated that the addition of DDDT could improve the oxidation resistance of the electrolyte.

**Figure 4:**
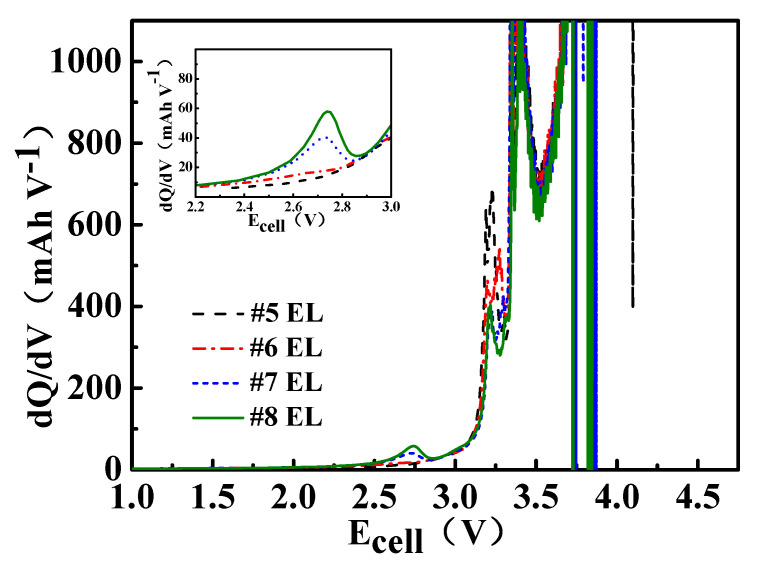
The dQ/dV plots of the assembled full cell.

The dQ/dV curve of the full battery containing 1–2 wt.% DDDT electrolyte shows a characteristic peak at around 2.8 V, which is lower than the reference group, indicating that the addition of DDDT is more beneficial than the addition of EC to the formation of the protective film on the negative electrode (Figure 4).

**Figure 5:**
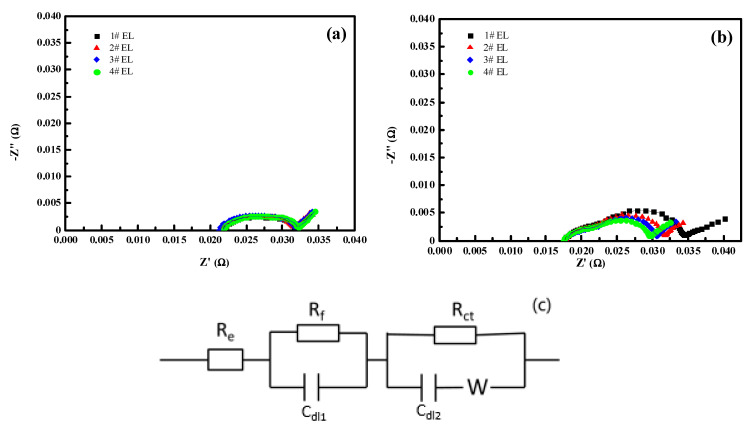
Representative EIS Nyquist plots and fitting curves of the two half-cells prepared with different electrolyte models (#1–#4 EL) after one cycle at 0.2 C: (**a**) graphite/Li; (**b**) LiCoO_2_/Li; (**c**) equivalent circuit model

Figure 5 shows the AC impedance data and the equivalent circuit model of LiCoO_2_/Li and graphite/Li, and Table 3 shows the fitting experimental results in different electrolytes (#1–#4 EL). The pattern in the impedance spectra is explained by an equivalent circuit diagram (Figure 5c). The R_e_ represents bulk resistance, which indicates the ohmic resistance of the electrolyte and electrodes. R_f_ and C_dl1_ are the charge-transfer resistance and the double-layer capacitance between the electrolyte and lithium metal corresponding to the semicircle at high frequencies, respectively. R_ct_ and C_dl2_ are the charge-transfer resistance and the double-layer capacitance between the electrolyte and cathode corresponding to the semicircle at medium frequencies, respectively. When the capacitances of the electrical double layer and the film are close to each other, the two semicircles will be merged into one semicircle in impedance spectra. *W* is the Warburg impedance related to the diffusion of lithium ions in the electrode, which is indicated by a slopping line at low frequencies. The combination of Rct and *W* is called faradic impedance, which reflects the kinetics of the cell reactions. The charge transfer (R_ct_) resistance of LiCoO_2_/Li decreased with an increase in the addition amount of DDDT, while no significant differences in the passivation film (R_f_) resistance of graphite/Li could be observed. The decomposition products would be accumulated on the electrodes, resulting in poor electrical contact and remarkable increase of resistance. Obviously, the full cells with DDDT additive in the electrolytes exhibited relatively lower resistance after storage and cycling tests, and we proposed that a newly generated interfacial film guaranteed the higher stability due to the possible decomposition reactions toward the electrolyte being efficiently inhibited. The degeneration of the electrode material could be minimized during the cycling and storage processes, and the parasitic interfacial reactions would be greatly reduced. The AC impedance tests of the half-cells reflected that the DDDT additive could facilitate the formation of the protective layers on the positive electrodes. The results were consistent with the relatively more excellent cycling stability and storage performances of the cells.

### 3.2. Electrical Performance of DDDT in Batteries Fully Charged to 4.5 V

#### 3.2.1. Effect of DDDT on DC Impedance

**Figure 6:**
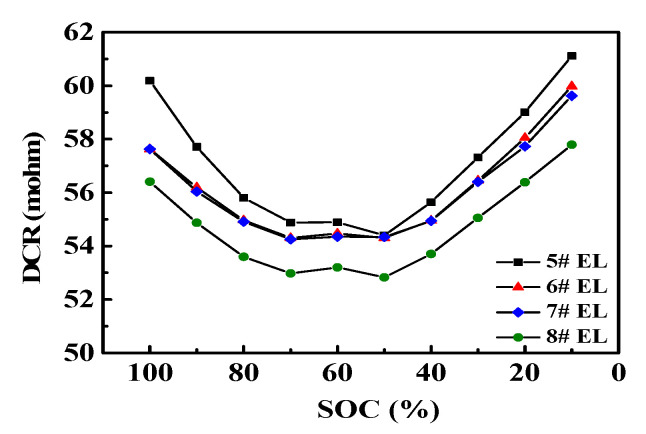
Discharge capacity retention (DCR) discharge at room temperature.

The results of DCR discharge at room temperature indicated that the DC internal resistance decreased with an increase in the addition amount of DDDT in the electrolyte (Figure 6). It is obvious that the smallest DC resistance could be obtained by the addition of 2 wt.% DDDT into the electrolyte.

#### 3.2.2. Effect of DDDT Additive on High-Temperature Storage

**Figure 7:**
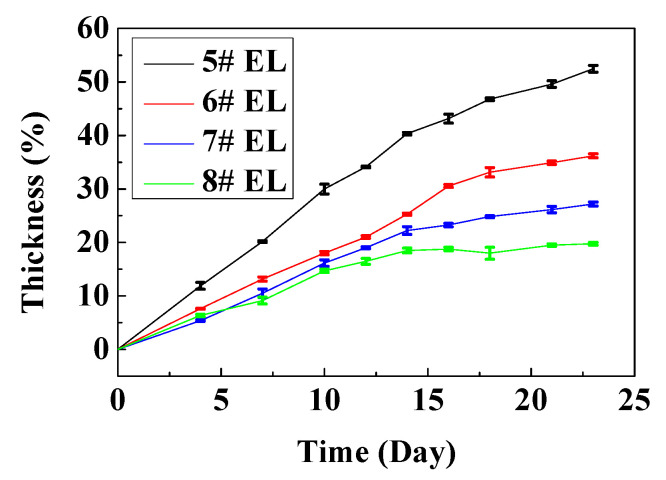
High-temperature storage test of the full battery at 60 °C.

The additive DDDT could significantly improve the high-temperature storage performance of the cells. The cells were stored at 60 °C for 23 days, and the thickness increased by 19.8%, 27.3%, and 36.2% when the amount of DDDT added into the electrolyte was 2 wt.%, 1 wt.%, and 0.5 wt.%, respectively. The thickness of the cell with the base electrolyte even increased by up to ~50%, indicating the addition of the appropriate amount of DDDT into the electrolyte was beneficial to the high-temperature storage performance of the cells (Figure 7). The thickness of the battery in 60 °C storage reflected the differences between positive electrode and electrolyte with and without DDDT, and the thickness of electrolyte with 2% DDDT was the smallest in electrolyte groups, reflecting that 2% DDDT can improve the stability of the positive electrode.

**Figure 8:**
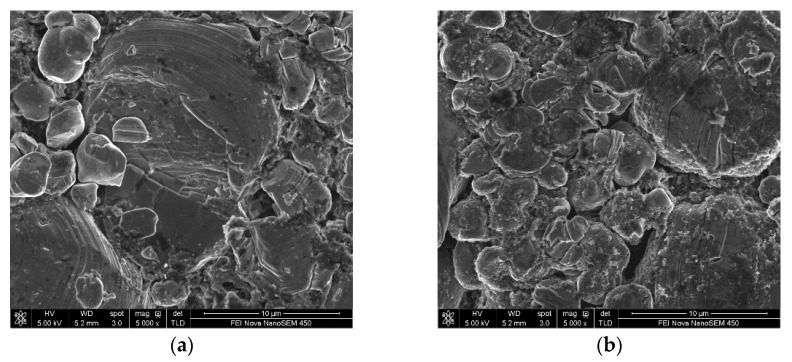

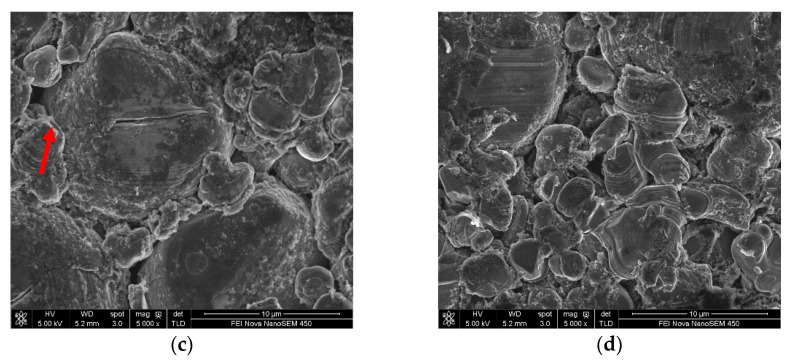
Morphological changes of the electrodes in different electrolyte groups before and after high-temperature (60 °C) storage: (**a**) in #5 EL before storage; (**b**) in #8 EL before storage; (**c**) in #5 EL after storage (the red arrow indicates the obvious cracks presented on the LiCoO_2_ particles in the base electrolyte without additive DDDT); (**d**) in #8 EL after storage.

The surface morphological changes of the electrodes (in #5 EL or in #8 EL) stored at 60 °C for 23 days were further investigated (Figure 8). It can be seen that obvious cracks are presented on the LiCoO_2_ particles in the base electrolyte without additive DDDT, while the surface of the LiCoO_2_ particles in the 2% DDDT electrolyte group was uniformly covered with a protective layer without any cracks. The results were consistent with the high-temperature (60 °C) storage performance of the full battery in different electrolytes (Figure 7). Table 4 shows the surface elemental distributions of the electrodes after the full battery was stored at 60 °C for 23 days. Sulfur could be detected on both the positive and the negative electrodes of the battery groups with additive DDDT, confirming the sulfide generated on their surfaces. The difference in the elemental distribution of cobalt (Co) on the negative electrode surface indicated that DDDT could be used as a positive electrode protection additive to prevent cobalt leaching and reduce the possible deposition on the negative electrode.

**Figure 9:**
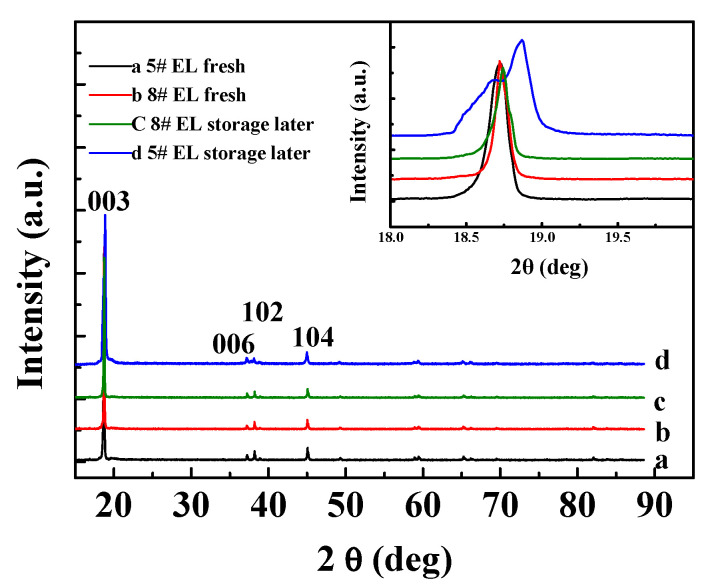
XRD characterization of the positive electrodes before and after high-temperature storage in different electrolyte groups (the inset illustrates the narrow scan of 003).

Figure 9 shows the structural changes of the positive electrodes in #5 EL and #8 EL groups before and after 60 °C storage for 23 days. No obvious differences could be observed for the (006), (102), and (104) peaks of XRD curves, but the 003 peak of the cell group (the insert illustrated in Figure 9) without DDDT additive was split, while the cell group with 2 wt.% DDDT electrolyte after high-temperature storage showed no significant changes in the peak shape in comparison with that before high-temperature storage. This is because the crystal structure of the positive electrode tended to be destroyed under high voltage and high temperature, and the addition of DDDT into the electrolyte could prevent the positive electrode from damage during the high-temperature storage.

#### 3.2.3. Effect of DDDT Additive on Cycle Performance

**Figure 10:**
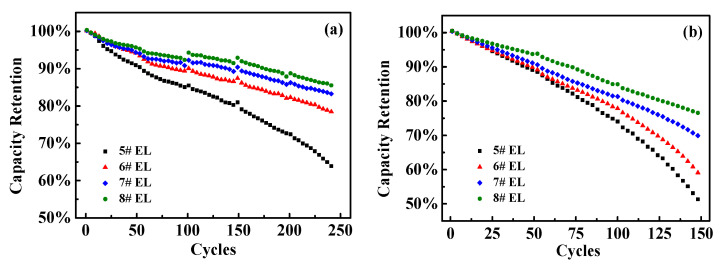
Capacity retention rates of the full batteries in different electrolytes: (**a**) 25 ℃; (**b**) 45 ℃.

DDDT might have a significant impact on the cycle performance of the battery (Figure 10). After 250 cycles at room temperature, the capacity retention rate remained about 77.7% for the battery in #6 electrolyte group (containing 0.5 wt.% DDDT), while the capacity retention rate in #8 electrolyte group (containing 2 wt.% DDDT) remained up to 84.5%. In contrast, for the battery with the base electrolyte #5 group without additive DDDT electrolyte, the capacity retention rate remained only about 62.1%. After 150 cycles at 45 °C, the capacity retention rate of the battery with the #8 electrolyte group (containing 2 wt.% DDDT) remained around 76.4%, while the battery with the base electrolyte #5 group had a relatively lower value of 52.5%.

**Figure 11:**
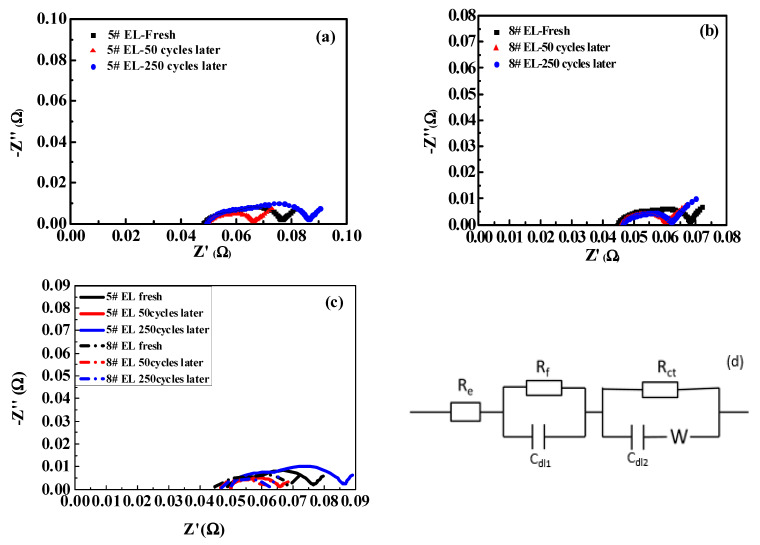
Representative EIS Nyquist plots measured and the fitting curves before and after cycling in different electrolyte models: (**a**) #5 EL (without additive DDDT); (**b**) #8 EL (containing 2 wt.% additive DDDT); (**c**) fitting curves of #5 and #8 EL; (**d**) equivalent circuit model.

In order to further reveal the underlying mechanisms of the improved cycle performance of the battery with DDDT additive, AC impedance analyses were performed. Figure 11 shows the AC impedance spectra before and after cycle tests. Table 5 shows the fitted data of the R_ct_ and R_f_ resistances. From the fitted data, the R_f_ and the R_ct_ resistances of the battery with 2 wt.% DDDT additive before cycle tests were slightly lower than those of the reference group without additive DDDT. The R_f_ and the R_ct_ resistances after 50 cycle tests were lower than previous data due to the improved infiltration of electrolyte in the pole piece, and the SEI film formed on the electrode was more stable after cycling over a period of time. As the number of cycle tests increased, the R_f_ and the R_ct_ resistances increased more significantly. After 200 cycle tests, the R_f_ and the R_ct_ resistances of the battery with base electrolyte increased to 22.46 and 14.05 mΩ, which were much higher than the original values of 17 and 10.2 mΩ, respectively. In contrast, the R_f_ and the R_ct_ resistances of the battery 2 wt.% DDDT additive only decreased to 11.43 mΩ and 3.49 mΩ compared with the original values of 13.59 and 9.02 mΩ, respectively. The results again confirmed that the protective layer formed by DDDT was beneficial in reducing the possible side reactions, thereby greatly reducing the AC impedance.

**Figure 12:**
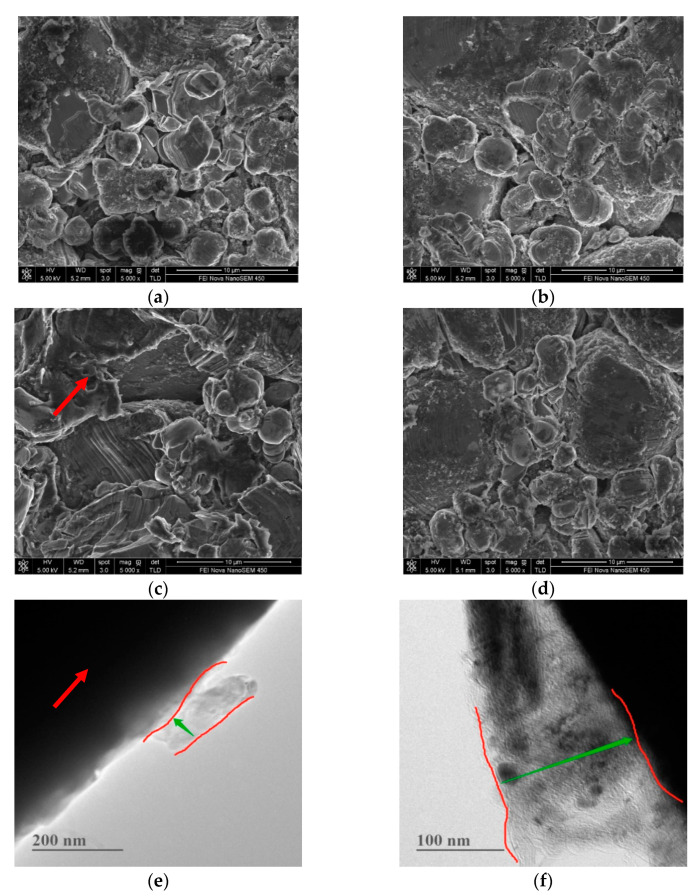
Morphological changes of electrode materials on cathodes with different electrolytes before and after cycle tests: (**a**) SEM image in #5 EL before cycling; (**b**) SEM image in #8 EL before cycling (the red arrow indicates the obvious cracks presented on the LiCoO_2_ particles in the base electrolyte without additive DDDT); (**c**) SEM image in #5 EL after cycling; (**d**) SEM image in #8 EL after cycling; (**e**) TEM image in #5 EL before cycling (nanoscale SEI layer was formed); (**f**) TEM image in #8 EL before cycling (the SEI layer formed was thicker than that in #5 EL).

**Figure 13:**
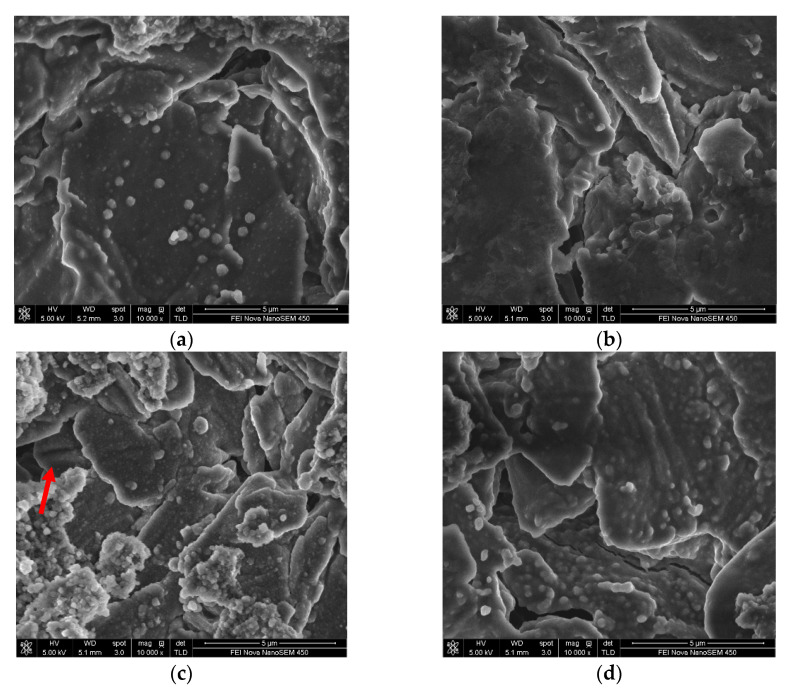
Morphological changes of electrode materials on anodes with different electrolytes before and after cycle tests: (**a**) #5 EL before cycling; (**b**) #8 EL before cycling; (**c**) #5 EL after cycling; (**d**) #8 EL after cycling.

Figure 12 and Figure 13 show the changes in the surface morphologies of the positive and negative electrodes with different electrolytes before and after 250 cycle tests. After 250 cycles of high-temperature cycling, cracks could be found for the LiCoO_2_ particles with the base electrolytes without any additive. When 2 wt.% DDDT was added into the electrolyte, the interface seemed more normal, again confirming that the DDDT additive had a significant protective effect on the positive electrode at a high voltage of 4.5 V (Figure 12a–d). The morphological changes of the negative electrode with DDDT additive were also relatively more unobvious than those of the negative electrode with the base electrolyte (Figure 13). TEM analyses further indicated that nanoscale SEI layers were formed, and the SEI layer formed in #8 EL was thicker than that in #5 EL (Figure 12e,f). The obtained results were consistent with the cycle performance tests of the battery, indicating that the DDDT additive had an improvement effect in both the positive and the negative electrodes.

**Figure 14:**
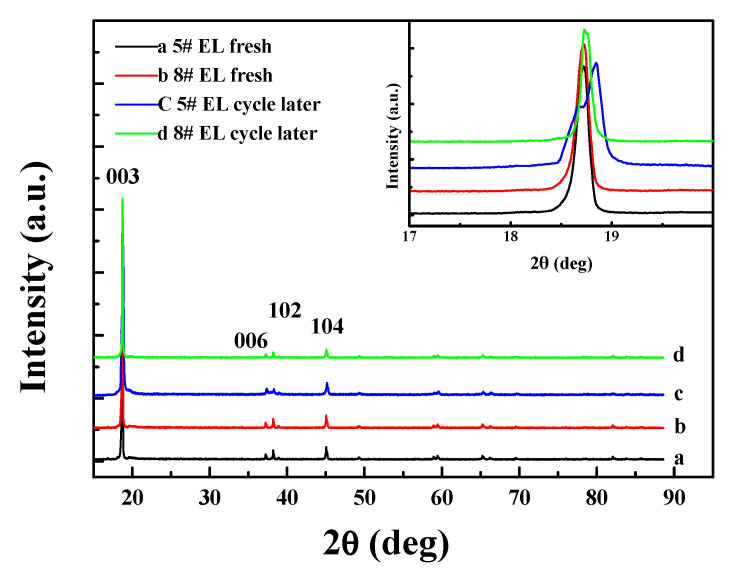
XRD characterizations for the positive electrodes in different electrolyte groups before and after cycle tests (The inset illustrates the narrow scan of 003).

Figure 14 shows the structural changes of the positive electrode before and after the cycling at 45 °C for the batteries with #5 and #8 electrolyte groups. No obvious differences could be observed in the (006), (102), and (104) peaks of XRD curves, but the 003 peak of the positive electrode without DDDT additive was split, and the electrode with 2 wt.% DDDT additive after storage showed no significant changes in the peak shape in comparison with that before cycling. This was because the positive electrode crystal structure was easily destroyed under high voltage and high temperature, and the addition of DDDT undoubtedly could protect or stabilize its structure.

**Figure 15:**
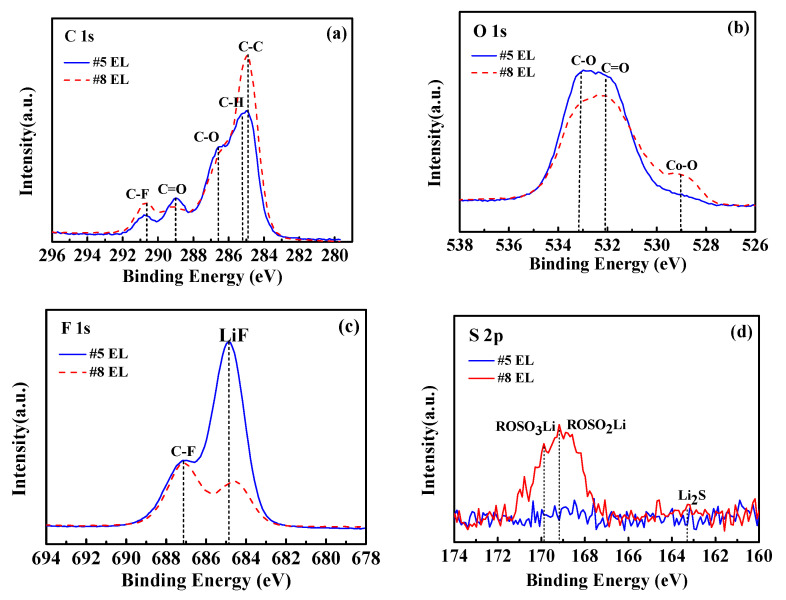
XPS spectra of the LiCoO_2_ cathodes cycled in the electrolyte without DDDT additive and in 2 wt.% DDDT after 150 cycles at 45 °C: (**a**) C 1s; (**b**) O 1s; (**c**) F 1s; (**d**) S 2p.

To assess the composition changes of the electrode surfaces cycled in the base and DDDT-containing electrolytes, XPS analyses of the cycled LiCoO_2_ and graphite electrodes after 150 cycles at 45 °C were performed. The XPS spectra of the cycled LiCoO_2_ cathode elucidated the effect of the DDDT additive on the CEI composition (Figure 15). In the C 1s spectra (Figure 15a), the C–C (284.9 eV) and C–H (285.3 eV) peaks of the cathode from the cells cycled in the electrolyte containing DDDT retained higher intensity than the cell cycled in the electrolyte without DDDT. The severe decomposition of electrolytes without DDDT during the cycling process was also confirmed by the C 1s spectra. For the O 1s spectra (Figure 15b), the obtained results of the C–O and C=O peaks were consistent with the C 1s peak analyses. These results could be compared with the intensity of the C–O (286.6 eV) and C=O (289.0 eV) peaks obtained for the cycled cathode in the electrolyte containing DDDT additive, which belonged to the decomposition products including ROCO_2_Li and polycarbonates [33]. Moreover, the Co–O peak of the cathode with DDDT additive was more obvious, indicating that the addition of DDDT promoted the formation of the thinner interfacial layer which relieved the severe oxidative decomposition of the electrolyte and reduced the coverage of decomposition products on the cathode surface. The cycled cathode in the electrolyte without DDDT possessed higher C–O and C=O intensities, indicating that the addition of DDDT additive can relieve the severe oxidative decomposition of the electrolyte on the cathode surface during the cycling process.

Due to the interactions between LiCoO_2_ and PVDF, Li–F (684.9 eV) and C–F (687.2 eV) peaks appeared (Figure 15c). In addition, the intensity of Li–F peak for the cathode in the DDDT-containing electrolyte was much lower than that in the electrolyte without additive, further indicating that the cathode surface in the electrolyte containing DDDT was covered with fewer electrolyte decomposition products, which was consistent with the results of the C–O and C=O peak analyses.

In the S 2p spectra (Figure 15d), no peak could be observed for the cycled cathode in the electrolyte without DDDT, while the peak corresponding to Li_2_S could be detected in the case in the electrolyte containing DDDT, indicating that the addition of DDDT promoted the formation of sulfide on the cathode [29]. For S 2p spectra of the cathode in #8 EL, the peak at 169.2 eV corresponds to ROSO_2_Li, and the peaks at 169.2 and 163.3 eV belong to ROSO_3_Li and Li_2_S, respectively, indicating the additive DDDT was preferentially oxidized to facilitate the formation of the SEI on the cathode.

**Figure 16:**
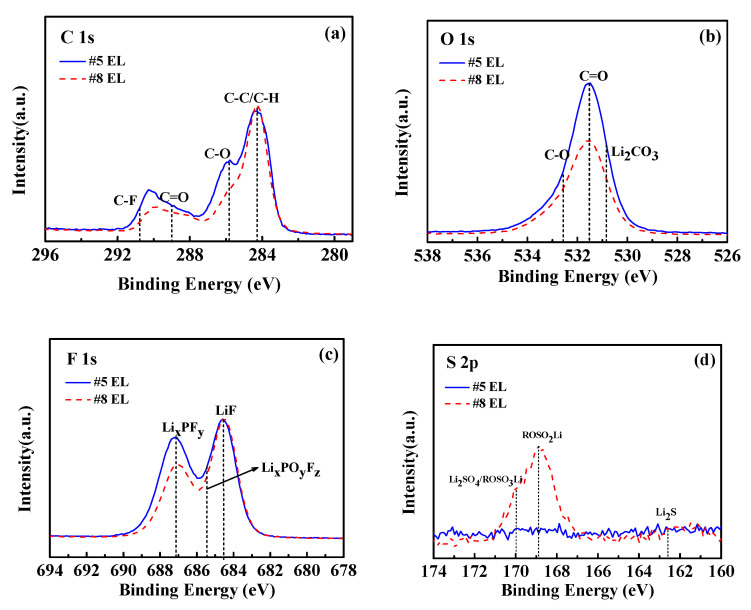
XPS spectra of the graphite anodes cycled in the electrolytes without DDDT additive and in 2 wt.% DDDT after 150 cycles at 45 °C: (**a**) C 1s; (**b**) O 1s; (**c**) F 1s; (**d**) S 2p.

Furthermore, the changes in the XPS patterns of the graphite electrodes in the electrolytes without DDDT and with DDDT additive were confirmed (Figure 16). In the C 1s photoelectron spectra, the peak of the C–C can be attributed to the graphite, and the peaks of C–O and C=O are assigned to the carbonate species such as RCH_2_OCO_2_Li due to the reductive decomposition of the electrolyte. In the O 1s spectra, the intensities of the C=O and C–O peaks for the graphite surface decreased when DDDT was added into the electrolyte compared with the graphite surface with the base electrolyte, indicating that the reductive decomposition of the electrolyte was alleviated and that the DDDT additive facilitated the formation of a passivation layer on the graphite surface. In addition, the graphite anode cycled in the electrolyte containing DDDT showed the lowest Li_2_CO_3_ peak intensity, suggesting less electrolyte was decomposed when the DDDT was added. In the F 1s photoelectron spectra, the Li_x_PO_y_F_z_, Li_x_PF_y_, and LiF would be generated from the decomposition and hydrolysis of LiPF_6_. The peak intensities of Li_x_PO_y_F_z_ and Li_x_PF_y_ from the graphite surface without DDDT were stronger than those cycled in the DDDT-containing electrolytes, which suggests that the superior SEI layer promoted by the additive can effectively relieve the LiPF_6_ decomposition on the anode. Furthermore, in the S 2p photoelectron spectra, sulfur-containing species were formed on the graphite surface with the DDDT additive, further indicating that the DDDT additive might have a reduction reaction on the graphite surface. Sulfur-containing species such as ROSO_2_Li and Li_2_SO_4_ could alleviate the reductive decomposition of electrolytes and promote the diffusion of lithium ions at the electrode–electrolyte interface, and the results were consistent with the dQ/dV analyses [34,35,36].

## 4. Conclusions

For the LiCoO_2_ system, the stability of the battery system deviates significantly as the voltage increases. Meanwhile, the impedance increases obviously and the storage and cycle performances deteriorate. To prevent side reactions and improve the high-pressure/high-temperature performance and cycle performance of the system, the addition of DDDT to the conventional carbonate solvent system was investigated. Protective layers could be effectively formed on the LiCoO_2_ or the graphite electrode surfaces, and the high-temperature storage performance of the battery was significantly improved. From the dQ/dV plots and AC impedance tests, the most appropriate addition amount of DDDT into the electrolyte was 2 wt.%. SEM, TEM, XRD, and XPS analyses confirmed the formation of stable nanoscale SEIs on both the positive and negative electrodes. LiCoO_2_ with 2 wt.% DDDT additive exhibited more excellent high-temperature Li-storage performance, controlled swelling behavior, and cycling performance.

## Figures and Tables

**Table 1:** Compositions of electrolytes #1–#4.

Code	Solvent		Additive (wt.%)	Lithium Salt (mol/L)
	EC	EMC	DDDT	LiPF6
**#1**	30	70	0	1.15
**#2**	30	70	0.5	1.15
**#3**	30	70	1	1.15
**#4**	30	70	2	1.15

**Table 2:** Compositions of electrolytes #5–#8.

Code	Solvent				Additive (wt.%)	Lithium Salt (mol/L)
	EC	PC	DEC	PP	DDDT	LiPF6
**#5**	20	10	30	40	0	1.15
**#6**	20	10	30	40	0.5	1.15
**#7**	20	10	30	40	1	1.15
**#8**	20	10	30	40	2	1.15

**Table 3:** The EIS fitting data of the coin half-cells.

Electrolyte Model	Graphite/Li		LiCoO_2_/Li	
	R_f_ (mΩ)	R_ct_ (mΩ)	R_f_ (mΩ)	R_ct_ (mΩ)
**#1 EL**	4.07	5.62	4.4	11.95
**#2 EL**	4.11	5.53	4.09	9.52
**#3 EL**	4.21	5.69	4.07	8.39
**#4 EL**	3.99	5.63	3.76	7.15

**Table 4:** Surface elemental distributions of the electrodes before and after high-temperature storage.

Elements		#5 EL		#8 EL	
		Before Storage (ppm)	After Storage (ppm)	Before Storage (ppm)	After Storage (ppm)
**Cathode**	C	18.9	16	17.4	15.6
	O	26.3	27.74	25.46	26.1
	Co	51.82	50	52.72	52.95
	P	0.34	0.56	0.44	0.25
	Al	0.42	0.36	0.39	0.44
	S	/	/	0.12	0.57
	F	2.22	5.34	3.47	4.09
**Anode**	C	74.89	30.07	76.18	48.59
	O	10.2	5.94	12.41	13.9
	P	0.59	0.76	0.54	0.69
	Cu	0.9	0.81	0.92	0.89
	S	/	/	0.14	0.31
	F	13.42	37.32	9.81	23.52
	Co	/	25.1	/	12.1

**Table 5:** Fitted data of the EIS Nyquist plots.

Electrolyte Code	Newly Prepared Cell		After 50 Cycles		After 250 Cycles	
	R_f_ (mΩ)	R_ct_ (mΩ)	R_f_ (mΩ)	R_ct_ (mΩ)	R_f_ (mΩ)	R_ct_ (mΩ)
**#5**	10.2	17	4.09	11.34	14.05	22.46
**#8**	9.02	13.59	3.43	9.81	3.49	11.43

## Data Availability

The data presented in this study are available on request from the first author (X.-Q.L., E-mail: xqliao@highpowertech.com).

## References

[1] Huo H., Xing Y., Pecht M., Züger B.J., Khare N., Vezzini A. (2017). Safety Requirements for Transportation of Lithium Batteries. Energies.

[2] Williard N., Hendricks C., Sood B., Chung J.S., Pecht M. (2016). Evaluation of Batteries for Safe Air Transport. Energies.

[3] Song Y., Liu D., Hou Y., Yu J., Peng Y. (2018). Satellite lithium-ion battery remaining useful life estimation with an iterative up-dated RVM fused with the KF algorithm. Chin. J. Aeronaut..

[4] Levchenko I., Xu S., Mazouffre S., Lev D., Pedrini D., Goebel D., Garrigues L., Taccogna F., Bazaka K. (2020). Perspectives, frontiers, and new horizons for plasma-based space electric propulsion. Phys. Plasmas.

[5] Levchenko I., Xu S., Wu Y.-L., Bazaka K. (2020). Hopes and concerns for astronomy of satellite constellations. Nat. Astron..

[6] Iclodean C., Varga B., Burnete N., Cimerdean D., Jurchiş B. (2017). Comparison of different battery types for electric vehicles. IOP Conf. Ser. Mater. Sci. Eng..

[7] Han G.B., Ryou M.H., Cho K.Y., Lee Y.M., Park Y.K. (2020). Effect of succinic anhydride as an electrolyte additive on electro-chemical characteristics of silicon thin-film electrode. J. Power Sources.

[8] Kang S.-H., Kempgens P., Greenbaum S., Kropf A.J., Amine K., Thackeray M.M. (2007). Interpreting the structural and electrochemical complexity of 0.5Li_2_MnO_3_·0.5LiMO_2_ electrodes for lithium batteries (M = Mn_0.5−x_Ni_0.5−x_Co_2x_, 0 ≤ x ≤ 0.5). J. Mater. Chem..

[9] Zhou F., Zhao X.M., Bommel A.V., Xia X., Dahn J.R. (2011). Comparison of Li [Li_1/9_Ni_1/3_Mn_5/9_] O_2_, Li [Li_1/5_Ni_1/5_Mn_3/5_] O_2_, LiNi_0.5_Mn_1.5_O_4_, and LiNi_2/3_Mn_1/3_O_2_ as high voltage positive electrode materials. J. Electrochem. Soc..

[10] Meng X., Dou S., Wang W.-L. (2008). High power and high capacity cathode material LiNi_0.5_Mn_0.5_O_2_ for advanced lithium-ion batteries. J. Power Sources.

[11] Yazami R., Ozawa Y., Gabrisch H., Fultz B. (2004). Mechanism of electrochemical performance decay in LiCoO_2_ aged at high voltage. Electrochim. Acta.

[12] Kim Y., Veith G.M., Nanda J., Unocic R.R., Chi M., Dudney N.J. (2011). High voltage stability of LiCoO_2_ particles with a nano-scale Lipon coating. Electrochim. Acta.

[13] Yang L., Markmaitree T., Lucht B.L. (2011). Inorganic additives for passivation of high voltage cathode materials. J. Power Sources.

[14] Xu K., Ding S.P., Jow T.R. (1999). Toward Reliable Values of Electrochemical Stability Limits for Electrolytes. J. Electrochem. Soc..

[15] Pei A., Zheng G., Shi F., Li Y., Cui Y. (2017). Nanoscale Nucleation and Growth of Electrodeposited Lithium Metal. Nano Lett..

[16] Jiao S., Ren X., Cao R., Engelhard M.H., Liu Y., Hu D., Mei D., Zheng J., Zhao W., Li Q. (2018). Stable cycling of high-voltage lithium metal batteries in ether electrolytes. Nat. Energy.

[17] Yoshida K., Nakamura M., Kazue Y., Tachikawa N., Tsuzuki S., Seki S., Dokko K., Watanabe M. (2011). Oxidative-stability enhancement and charge transport mechanism in glymeàlithium salt equimolar complexes. J. Am. Chem. Soc..

[18] Amanchukwu C.V., Yu Z., Kong X., Qin J., Cui Y., Bao Z. (2020). A new class of ionically conducting fluorinated ether electro-lytes with high electrochemical stability. J. Am. Chem. Soc..

[19] Xu K., Angell C.A. (2002). Sulfone-based electrolytes for lithium-ion batteries. J. Electrochem. Soc..

[20] Sun X.G., Angell C.A. (2005). New sulfone electrolytes for rechargeable lithium batteries: Part I. Oligoether-containing sulfones. Electrochem. Commun..

[21] Lewandowski A., Świderska-Mocek A. (2009). Ionic liquids as electrolytes for Li-ion batteries—An overview of electrochemical studies. J. Power Sources.

[22] Guerfi A., Dontigny M., Charest P., Petitclerc M., Lagacé M., Vijh A., Zaghib K. (2010). Improved electrolytes for Li-ion batteries: Mixtures of ionic liquid and organic electrolyte with enhanced safety and electrochemical performance. J. Power Sources.

[23] Xu M., Liu Y., Li B., Li W., Li X., Hu S. (2012). Tris (pentafluorophenyl) phosphine: An electrolyte additive for high voltage Li-ion batteries. Electrochem. Commun..

[24] von Cresce A., Xu K. (2011). Electrolyte additive in support of 5 V Li ion chemistry. J. Electrochem. Soc..

[25] Lee J.-N., Han G.-B., Ryou M.-H., Lee D.J., Song J., Choi J.W., Park J.-K. (2011). N-(triphenylphosphoranylidene) aniline as a novel electrolyte additive for high voltage LiCoO_2_ operations in lithium ion batteries. Electrochim. Acta.

[26] Zuo X., Fan C., Xiao X., Liu J., Nan J. (2012). High-voltage performance of LiCoO_2_/graphite batteries with methylene me-thanedisulfonate as electrolyte additive. J. Power Sources.

[27] Wang Z., Dupré N., Lajaunie L., Moreau P., Martin J.-F., Boutafa L., Patoux S., Guyomard D. (2012). Effect of glutaric anhydride additive on the LiNi_0.4_Mn_1.6_O_4_ electrode/electrolyte interface evolution: A MAS NMR and TEM/EELS study. J. Power Sources.

[28] Tarnopolskiy V., Kalhoff J., Nádherná M., Bresser D., Picard L., Fabre F., Rey M., Passerini S. (2013). Beneficial influence of suc-cinic anhydride as electrolyte additive on the self-discharge of 5 V LiNi_0.4_Mn_1.6_O_4_ cathodes. J. Power Sources.

[29] Kubota T., Ihara M., Katayama S., Nakai H., Ichikawa J. (2012). 1,1-Difluoro-1-alkenes as new electrolyte additives for lithium ion batteries. J. Power Sources.

[30] Li Y., Wang X., Dong S., Chen X., Cui G. (2016). Recent Advances in Non-Aqueous Electrolyte for Rechargeable Li-O_2_ Batteries. Adv. Energy Mater..

[31] Aravindan V., Cheah Y.L., Ling W.C., Madhavi S. (2012). Effect of LiBOB Additive on the Electrochemical Performance of LiCoPO_4_. J. Electrochem. Soc..

[32] Lee S.H., Hwang J.Y., Park S.J., Park G.T., Sun Y.K. (2019). Adiponitrile (C_6_H_8_N_2_): A new Bi-functional additive for high-performance Li-metal batteries. Adv. Funct. Mater..

[33] Yang T., Wang W., Li S., Lu J., Fan W., Zuo X., Nan J. (2020). Sulfur-containing C_2_H_2_O_8_S_2_ molecules as an overall-functional electrolyte additive for high-voltage LiNi_0.5_Co_0.2_Mn_0.3_O_2_/graphite batteries with enhanced performance. J. Power Sources.

[34] Zuo X., Deng X., Ma X., Wu J., Liang H., Nan J. (2018). 3-(Phenylsulfonyl) propionitrile as a higher voltage bifunctional electro-lyte additive to improve the performance of lithium-ion batteries. J. Mater. Chem. A.

[35] Li S.-C., Yu J.-G. (2020). A well-designed CoTiO3 coating for uncovering and manipulating interfacial compatibility between LiCoO_2_ and Li_1.3_Al_0.3_Ti_1.7_(PO_4_)_3_ in high temperature zone. Appl. Surf. Sci..

[36] Madec L., Xia J., Petibon R., Nelson K.J., Sun J.P., Hill I.G., Dahn J.R. (2014). Effect of sulfate electrolyte additives on LiNi_1/3_Mn_1/3_Co_1/3_O_2_/graphite pouch cell lifetime: Correlation between XPS surface studies and electrochemical test results. J. Phys. Chem. C.

